# Housing Orientations and Needs of Above-Average Length of Stay Hospitalized Psychiatric Patients

**DOI:** 10.3389/fpsyt.2020.00231

**Published:** 2020-04-07

**Authors:** Filippo Rapisarda, Amélie Felx, Stéphane Gagnon, Luigi De Benedictis, André Luyet, Marc Boutin, Marc Corbière, Alain Lesage

**Affiliations:** ^1^Sociosfera ONLUS, Seregno, Italy; ^2^Institut universitaire en santé mentale de Montréal (IUSMM), Centre intégré universitaire de santé et de services sociaux (CIUSSS) de l’Ouest-de-l’Île-de-Montréal, Montréal, QC, Canada; ^3^Douglas Mental Health University Institute, Centre intégré universitaire de santé et de services sociaux (CIUSSS) de l’Ouest-de-l’Île-de-Montréal, Montréal, QC, Canada; ^4^Département d’éducation et pédagogie, Université du Québec à Montréal, Montréal, QC, Canada

**Keywords:** housing, residential care adults, needs, mental health, hospitalization

## Abstract

A small number of severely and persistently mentally ill in-patients awaiting residential or long-stay facilities represent an obstacle to the efficient utilization of acute care beds. These facilities are costly and currently reputed to be contrary to recovery principles. In 2013, all acute psychiatric care wards in Montreal identified 194 in-patients who could be discharged to residential or long-term nursing care facilities. Program clinical professionals of regional residential facilities sent adapted standardized questionnaires to ward staff. Evaluators also collected the residential preferences of both staff and patients, and then made their own assessments. The 194 in-patients were mostly middle-aged single men. Over 80% had a psychosis diagnosis and half had judicial constraints. The staff evaluated that 71.1% could be discharged from hospital within 24 h. Of these, 55% could be referred to group resources with continuous 24 h, 7 days a week staff presence, 32% could be transferred to apartments with 7-day continuous or non-continuous staff presence, 12% could be transferred to institutional care and only 2% could be moved to an apartment of their own. Evaluator and ward staff residential preferences were highly similar, but differed with patient preferences, half of whom prefer their own apartment. Discrepancy between staff evaluations and patient preferences were higher for longer stay patients with more severe symptoms and comorbidity of personality disorders.

## Introduction

A balanced mental health care system for severely and persistently mentally ill patients in a resource-rich country like Canada includes several treatment and rehabilitation components: community mental health team (CMHT), intensive home care intensive home care (IHC)—including intensive case-management team (ICM) and assertive community treatment team (ACT), residential facilities and long-term hospitalization, forensic psychiatric beds, as well as occupational facilities (1). Over the past decades most industrialized countries have had similar experiences of downsizing or closing psychiatric hospital long-stay beds, and increasing acute care beds, CMHTs and residential facilities. This period has also been characterized by insufficient funding, trans institution to the judicial system and destitution into the streets of severely mentally ill patients and, more recently in Quebec, further cuts in CMHTs, acute care beds, and residential facilities due to budgetary constraints. In Canada, cuts in the mental health budget have been proportionately greater than in the UK or Australia that were considered by a recent Canadian senatorial committee as re-investing in their mental health systems based on needs (2).

The number of places for each element in a given catchment-area was estimated by Wing in the ‘90s in a seminal work for the Royal College of Psychiatrists, and also by Wing, Thornicroft and Brewin (3). Combining a bottom-up evaluation of acute care beds, in-patient needs, and staff preferences for clients of residential facilities in the east end of Montreal, the following benchmarks were suggested: acute care beds (18 per 100,000 inhabitants); intensive home care (about 250 places per 100,000 inhabitants); 131 residential places, 20 nursing homes beds, and 20 long-stay beds per 100,000 inhabitants (4). The estimates did not take into account homeless mentally ill patients concentrated downtown Montreal, or prison inmates with psychosis (5). The latter study estimated that 8% of Quebec prison inmates had previously received a diagnosis of schizophrenia while the yearly treated prevalence in the population is 0.4% (6).

In each jurisdiction, the number of each type of acute care beds, residential facilities, or assertive community treatment teams will depend on historical and professional culture, and pressure on each type of service will depend on the availability of other types of services, not to mention outright closure of existing facilities. Signs of pressure are abundant (7): Canadian urban emergency rooms being on permanent overcapacity protocols (8); hospital psychiatric wards being at 100% capacity or more (while an average of 85% would ensure better quality (9); a long waiting list for supervised residential settings or assertive community treatment teams (10). The flow of patients in acute care wards is particularly sensitive to the small number of patients with longer lengths of stay. In one east-end Montreal acute care ward study, 37% of the patients admitted for over one month accounted for 87% of all bed-days and 13% admitted for over 3 months accounted for 56% all bed-days (4). At the time, the needs of these patients deemed ready for discharge was mainly for intensive home care and, to a lesser extent, for further supervised residential settings like group residential and supervised apartments with staff on the premises (4, 11). The 2005–2010 Quebec mental health action plan for the severely mentally ill adopted a recovery-oriented philosophy calling for the development of intensive home care, reduction of supervised residential facilities below our estimates of needs (11) and patient-led intervention plans with the introduction of peer-support workers. However, in 2013, less than a third of ACTeams had been developed (and only about 28 in Montreal, and 30% for ICM places); half of the 135 trained peer-support workers found contractual employment in public services in Quebec (8 million inhabitants); cuts in residential facilities have continued in regions considered overcapacity [Centre national d'excellence en santé mentale (CNESM), personal communication. https://cnesm.org/].

The scope of the present study was to further document the needs of long-stay in-patients in psychiatric acute care wards. In 2013, the Montreal Regional Health and Social Services Agency (ASSSM) conducted a survey of all acute care wards in Montreal for patients awaiting residential placement. This occurred in the context where the Ministry of Health and Social Services investigated the ASSSM for failure to meet acute care bed ratios, for excessive over-48-h emergency rooms stays by psychiatric patients, for insufficient downsizing of residential facilities, for insufficient mental health budget transfers to community organizations, for critical incidents with a threatening homeless severely mentally ill patient being killed by police. A standardized procedure and questionnaires were used by professional clinicians with ward staff, that allow comparisons for patient clinical and social characteristics, and preferences for housing and support on a population basis, that would be of interest in other resource-rich countries.

## Methods

### Procedure

The project consisted of a cross-sectional survey that targeted two psychiatric hospitals with catchment-area acute care hospitalization responsibilities, nine general hospital psychiatric wards and one forensic mental health hospital all located in Montreal (total capacity at the time of the survey was 1,159 beds or 64 beds per 100,000 inhabitants). All patients with longer stay than one month, waiting for a place/bed in a community-based residential facility or likely to be referred to a residential facility upon discharge were considered eligible for the study. Patients in an acute stabilization phase, admitted for less than a month or in the provincial forensic psychiatric hospital were excluded.

For each patient, the hospital staff (mainly nursing staff) completed a questionnaire adapted from five existing instruments [Canadian Psychosocial Rehabilitation (PSR) Toolkit, Nottingham Acute Beds Utilisation Schedule (NABUS), Level of Care Survey (NYLOCS), Riverview Patient Inventory (RPI), Consumer Housing Preference Survey (CHPS)]. After completion, one or two staff members met two evaluators. Evaluators systematically reviewed the questionnaire answers with staff member(s) and asked questions on the patient’s strengths, interests, rehabilitation readiness, and social network. For each patient, the final question explored staff member perception of ideal services. Housing orientation was also assessed independently by the evaluators on the basis of all available information. Data were collected by 15 experienced clinicians (evaluators) between March and April 2013, including AF and SG.

Of the 270 users who were evaluated for the project, 57 (21.1%) were excluded from the present study because they came from a forensic psychiatric hospital, and a further 19 (7.0%) were excluded because they had been hospitalized for less than 30 days. As a result, the final sample consisted of 194 subjects.

### Instruments

A modified version of the RPI (12) was used to assess patients’ clinical needs. The RPI is a behavioral rating scale that can be feasibly used by mental health staff to assess patients’ clinical conditions over four different but interrelated dimensions: daily routines, psychological symptoms, social interaction, and aggressive behavior. The instrument was scale designed to assess problem behaviors that have an effect on treatment and community placement. It was developed to be a quick and convenient tool for nurses and other caregivers as a means of obtaining a comprehensive assessment. The RPI provides rapid assessment of a pertinent repertoire of behavioral difficulties and symptoms of psychiatric inpatients. Its administration requires little or no training. The scale appears to validly discriminate poorly functioning patients from higher functioning, less ill ones (12). To better estimate the level of clinical needs, the research team adopted the Trudel and Lesage version (13) that consists of 55 items and includes a fifth scale, labeled “Problems in Relation to Community Preparation”. Each item is scored on a 5-point scale (from 1, no problem, to 5, severe problem). This version has already been translated and adopted in French and English (14). Cronbach’s Alpha, computed from the select sample of 194 above-average-stay patients, was 0.94 for the total score and ranged from 0.86 (community preparation) to 0.75 (daily routine) for the subscales. Similar values were reported by Haley (12).

A modified version of the CHPS (15, 16) was used to assess staff and patient housing preferences. The original instrument consisted in 22 statements concerning the person’s current living situation, housing preference, and support services required to live in the preferred housing. Most questions have a response choice. The modified version had integrated the taxonomy of housing preferences first used by the authors when they modified and used the NABUS (11).

The physical problems scale of the Levels of care survey (NYLOCS) was used, as we did previously in a study of the discharge of long-stay ward inpatients (17, 18). This questionnaire developed for long-stay psychiatric inpatients and nursing homes residents comprises a series of physical and behavioral scales. In this study, we only used the physical autonomy items (i.e. walking ability).

The Psychosocial Rehabilitation Toolkit (14, 19) was adopted to collect sociodemographic data (age, sex, native language, and marital status), as well as education level, work history, residential history, and financial, legal, and diagnostic information (diagnosis and comorbidities).

Housing preferences by patient and staff were recorded according to the CHPS; evaluators translated current administrative and detailed types of ASSSM residential resources, described by Felx and colleagues (20) into CHPS categories.

### Data Analysis

Descriptive statistics were computed for a selection of variables covering socio-demographics, clinical evaluations, and suggested residential orientation. In order to compare evaluator and staff residential needs assessment with patient residential preferences, the CHPS and ASSSM scores were recoded using a common set of four categories:

- “own apartment”: CHPS “own apartment”, ASSSM “individual apartment”.
- “apartment with support”: CHPS “supervised apartment”, ASSSM “apartment with other tenants” and “congregate apartments”.
- group residence: CHPS “group residence”, “foster home”, “transitory residential resource”, ASSSM “group resource”, “forensic psychiatric group resource” and “dual diagnosis group resource”‘.
- institution: CHPS “hospital unit”, “nursing home”, ASSSM “institution”.

Residential assessment was cross tabulated with users’ choice, and Cohen’s Kappa index was computed to estimate the degree of agreement between evaluators and users and between staff and users. Furthermore, evaluators’ assessments and users’ residential preferences were combined and recoded into one binary variable, with 1 indicating agreement and 0 indicating disagreement, which made it possible to split the sample into two subgroups. Discrepancies between evaluators’ assessments and patients’ preferences were analyzed by comparing socio-demographic and clinical variables for two groups using chi square and Fischer’s exact test for categorical variables and t test for continuous variables. Variables with statistically significant comparisons were adopted as explanatory variables of a logistic regression model with agreement/disagreement variables as targets. Pseudo R square indexes (21, 22) were computed to test model fit properties. All analyses were done using IBM SPSS^®^ version 21.

## Results

### Sample Characteristics

**Tables 1** and **2** show the sociodemographic and clinical characteristics of the sample. Sample mean age was 46.4 (SD = 16.3) with a relevant number of young patients under 30 (20.1%) and older patients aged 65 or more (14.4%). Most of the patients were Caucasian (74.8%), lived alone (52.1%) or with relatives (26.8%) and had completed high school (69.6%). For most of them (73.7%) the primary source of income came from welfare and more than half (51.0%) were under legal constraint (public curatorship or a community treatment order).

**Table 1:** Socio-demographic variables of 194 acute care ward in-patients earmarked for residential/institutional placement.

	N	%
**Age**		
Age between 18 and 29	39	20.1%
Age 65 or more	28	14.4%
*Missing*	8	4.1%
**Sex**		
Male	130	67.0%
*Missing*	0	0.0%
**Ethnic origin**		
Caucasian (European, North-American)	144	74.2%
Antilles (Haiti, Jamaica)	23	11,9%
Other	27	13,9%
*Missing*	0	0.0%
**Civil status**		
Single, living alone	101	52.1%
Single, living with relatives	52	26.8%
Separated or divorced	26	13.4%
Married or civil union	12	6.2%
*Missing*	3	1.5%
**Education (highest completed)**		
Elementary	43	22.2%
High school	96	49.5%
College or higher	39	20.1%
*Missing*	16	8.2%
**Principal income source**		
Welfare	143	73.7%
Old age pension	31	16.0%
Other	17	8.8%
No income	3	1.5%
*Missing*	0	0.0%
**Legal status**		
Under legal restraint	99	51.0%
Ordinance	63	32.5%
Under curatorship	56	28.9%
Under the Administrative Tribunal of Quebec	54	27.8%
*Missing*	0	0.0%

**Table 2:** Clinical variables of acute care wards in-patients earmarked for residential/institutional placement.

	N	%
**Previous hospitalizations in the last 2 years**		
No previous hospitalizations	33	17.0%
At least one previous hospitalization	139	71.6%
Already in hospital for more than 2 years	16	8.2%
*Missing*	6	3.1%
**Length of the current hospitalization**		
≤ 3 months	68	35.1%
4 – 12 months	91	46.9%
More than 12 months	35	18.0%
*Missing*	0	0.0%
**Diagnosis (Axis 1)**		
Psychotic disorder	170	87.6%
Affective disorder	18	9.3%
Other	6	3.1%
*Missing*	0	0.0%
**Dual diagnosis**		
Personality disorder	50	25.8%
*Missing*	1	0.5%
Intellectual disability	19	9.8%
*Missing*	2	1.0%
Substance abuse	62	32.0%
*Missing*	1	0.5%
**Walking ability**		
Totally autonomous/without any problem	173	89.6%
Walks unsteadily or with a cane	11	5.7%
Walks with a walker or a wheelchair	7	3.6%
*Missing*	3	1.5%

More than two-thirds of the patients (71.6%) had been hospitalized in the last two years prior to the current hospitalization that; for 18.0% of the cases, lasted for more than one year. Psychotic disorders are the most common diagnoses (87.6%) in DSM-IV axis I, and co-morbidities with substance abuse (32.0%) and personality disorders (25.8%) were also common.

RPI assessment was performed for 172 patients (88.7%), results are displayed in **Table 3**. Mean RPI total score was 75.5 (95%CI:70.7–80.3), corresponding to a mild severity, lower than the tertiary psychiatric services very long-stay inpatient scores obtained by Petersen (14) (mean 101; 95%CI 98.2–105.4).

**Table 3:** Riverview Patient Inventory (RPI) Scores.

	Mean	SD
RPI Total score	75.5	34.1
RPI Daily routine	24.5	10.7
RPI Psychological symptoms	17.7	9.1
RPI Social interaction	10.1	6.0
RPI Aggressive behavior	7.4	6.2
RPI Community preparation	15.7	8.2

### Residential Needs Evaluation

Residential needs evaluation and patients’ preferences are displayed in **Table 4**. Patients’ preferences couldn’t be obtained for 19 subjects, and no missing data were found for evaluators and staff choice. Group resource was the most frequent residential solution proposed both by evaluators (52.0%) and by staff (67.4%) but not by patients who preferred a group resource only in 26.9% of cases. Patients’ first preference was the apartment option (65.2%) that, in most cases, (46.9%) was their own apartment.

**Table 4:** Patients and professional clinical evaluators or treating staff choice of residential/institutional services.

	Patient choice									
	Own		Apartment with support		Group		Institution		Total	
	**N**	**%**	**N**	**%**	**N**	**%**	**N**	**%**	**N**	**%**
**Evaluator choice**										
Own Apartment	13	7.4%	2	1.1%	4	2.3%	1	0.6%	20	11.4%
Apartment with on-site support	19	10.9%	8	4.6%	7	4.0%	3	1.7%	37	21.1%
Group resource	38	21.7%	16	9.1%	28	16.0%	9	5.1%	91	52.0%
Institution	12	6.9%	6	3.4%	8	4.6%	1	0.6%	27	15.4%
Total	82	46.9%	32	18.3%	47	26.9%	14	8.0%	175	100.0%
**Staff choice**										
Own Apartment	2	1.1%	1	0.6%	1	0.6%	0	0.0%	4	2.3%
Apartment with on-site support	8	4.6%	3	1.7%	3	1.7%	1	0.6%	15	8.6%
Group resource	60	34.3%	20	11.4%	32	18.3	6	3.4%	118	67.4%
Institution	12	6.9%	8	4.6%	11	6.3	7	4.0%	38	21.7%
Total	82	46.9%	32	18.3%	47	26.9%	14	8.0%	175	100.0%

Agreement between evaluators’ assessment and patients’ preferences occurred in 30.6% of cases, corresponding to a total lack of agreement (Kappa = 0.05). An even lower agreement rate (25.1%, Kappa = 0.03) was found between staff evaluation and patient preferences. Discrepancies were also found between staff and evaluators (43.2% Kappa = 0.06).

The logistic regression model of discrepancies between evaluators and patients found a slightly statistically significant effect of having a personality disorder (B = -4.03; Wald = 14.65 OR =.02), the length of the current hospitalization longer than 12 months (B= -.81; Wald = 4.41; OR =.45) and interaction between personality disorder and total RPI score (B = 0.32; Wald = 11.00; OR = 1.03). However, even though the model was able to correctly predict 65.7% of cases, fit indexes were low (Cox and Snell R square =.13; Nagelkerke R square =.18).

## Discussion

All long-stay inpatients in acute care wards in Montreal awaiting residential resources at the time of the survey (at the end of 2013), were indeed assessed independently by clinical professionals as requiring such resources. It represents about 7% of existing psychiatric residential resources in Montreal. Absence of a regular flow of patients from these resources results in waiting times, in more costly hospital wards, sclerosis of social and living skills, and less hope in recovery. The characteristics of these middle-aged men with months of hospitalization, psychosis, judicial constraints, of which a third have substance abuse problems, and a quarter have personality disorders, would qualify them as candidates for Assertive Community Treatment (ACT) or ICM in suitable housing. Indeed, such an arrangement would be preferred by half the patients. The discrepancy between staff assessment of higher intensity residential facilities and patient preferences is consistent with national and international residential preference surveys of severely mentally ill patients (23). If severity of symptoms and behaviors may justify why staff differ in preferring more supervised residential facilities, the statistical models demonstrating such effect in this sample did explain only a small part of the discrepancy. Similar needs for own apartment and support by intensive home care was found 15 years ago in a comparable study of acute care wards in the east end of Montreal (11). The resulting benchmarks were used by the Ministry of Health and Social Services in its 2005–2010 action plan (24) and again in its 2015–2020 plan (25), this time reducing by half its residential facilities benchmarks. On the other hand, these in-patients found in acute care wards differ in their lower incapacity, measured with the RPI, from long-stay inpatients of psychiatric hospitals who were successfully and mostly transferred to regional tertiary residential facilities or other supervised residential facilities (not their own apartments) in British Columbia in the last decade (14), or in Montreal two decades ago (18, 26) or those assessed in nursing homes in a Quebec region with no psychiatric hospital (13). Overall, the convergence of evidence from these studies and comparison with the level of incapacity of our patient sample, points to the patient’s choice being the most accurate assessment of residential services needs.

There are limitations associated with the design of this study in representing the need for residential resources. First, only in-patients earmarked for residential facilities were selected by staff. Secondly, our study did not allow us to compare short-stay patients versus long-stay. It could be hypothesized that long-stay patients receive less rehabilitation interventions addressing social functioning compared to their needs, and that factor could explain why they have longer stay in the hospital ward. Thirdly, the NABUS questionnaire was modified by the Agency from the original Montreal (4) study so as not to independently cover the need for intensive home care. Staff may have considered this option more often, even though it was not readily available in Montreal at the time of the study. Fourthly, patients’ perspective was not collected independently, which may have increased their rating of their own apartment. Fifthly, the absence of patient representatives or peer-support workers in the assessment team is a finding about the program evaluation reported by this study. It was a patient-centered but not a patient-led approach. Finally, a more complete needs assessment would also consider the homeless as well as severely mentally ill prison inmates.

The findings support the final report by a Ministry of Health and Social Services inspector for Montreal (26) which recommend giving priority to the development of intensive home care, both ACT and Intensive Case Management, up to 1,627 ACTeam places (about 100 per 100,000 inhabitants) and 6,000 ICM places (about 320 per 100,000 inhabitants), more collaboration with existing residential resources, and a 25% increase of existing residential resources. It also recognizes the need for nursing homes and the increased specialization of existing residential resources. The report remains silent on the Supplement to Rent (STR), which is surprising since, in a simulation of the number of places and people in need of specialist care in a balanced mental health care system for people with severe mental illness (SMI) (27), we demonstrated that the combination of ACT or ICM with STR would cost $9,000–$14,000 per person per year, while group resources now average $49,000 and supervised apartments with continuous day staff presence cost $19,000. The feasibility and value of the combination of ACT or ICM with STR for the most severely mentally ill in the community, namely homeless severely mentally ill patients, was demonstrated in the At Home project in five Canadian cities, including Montreal, that recruited homeless severely mentally ill patients through peer-support workers with lived experience of homelessness, and successfully offered these homeless patients access to own apartment with STR, and clinical support by an ACT-Team or ICM-Team (28, 29). A recent international consensus conference on transitions to community of services for the severely mentally ill warned to complete the deployment of flexible assertive community teams before decreasing further hospital and residential facilities beds (30).

Will priority be given to the development of such an approach which would fit the preference of in-patients awaiting resources, and to those currently in such resources who now consider this to be their choice (23, 28). It could be argued that the co-morbidity of psychosis with personality disorder may influence the choice of such patients; however, Côté and colleagues (31) showed that psychotic patients with personality disorder have higher social autonomy to which they could legitimately aspire, but not always recognized by staff. This may not translate to progressively closing existing residential facilities but adapting them to welcome more severely mentally ill patients, patients with judicial constraints or physical frailty, and specialized homes for Natives or youth with psychosis. A balanced mental health care system will always require an array of residential resources, some very highly staffed and with proper programming that represent alternatives to long-stay hospitals or former psychiatric hospitals such as the tertiary psychiatric residential facilities developed in British Columbia (32). The array of residential services will also include foster families, group homes, and congregated housing and apartments (28). Such facilities have been described and are reputed as less recovery-oriented, yet evidence shows that they represent the best choice for patients at a point in time in their recovery process (23, 33). Recovery-orientation is not linked to the type of residential facilities per se, but to programming, competence of staff and full participation of residents and their families (32, 34).

## Conclusion

Over half of the long-stay acute care wards in-patients in Montreal would prefer their own apartment with intensive home care. This would prove less costly than the group homes earmarked for most of them by ward and residential program professionals who evaluated their needs. Our findings also suggest that such evaluation of needs, treatment and rehabilitation shall involve peer-support workers like in the UK, alongside professional staff (35), to ensure a more efficient, patient-led and recovery-oriented system of mental health care for the severely and persistently mentally ill.

### Ethics

Given that this is secondary analysis of data from program evaluation, ethical evaluation was performed by the Mental Health Agency Directorate that commissioned the survey. The Mental Health Agency is regional governmental agency designed to manage and evaluate mental health services. Additional ethical evaluation was performed by the Head of professional services at the IUSMM responsible for the safe and confidential keeping of the data. Moreover, all the data obtained for the analysis was anonymous. Thus, given the aforementioned design of the study and the anonymity of the data collection, the Ethics Committee was only informed of the study, and gave formal approval in 2019. All the participants (or a legal representative) filled in an Informed Consent during the stay in the acute ward.

## Data Availability Statement

The datasets generated for this study are available on request to the corresponding author.

## Ethics Statement

Ethical review and approval was not required for the study on human participants in accordance with the local legislation and institutional requirements. The patients/participants provided their written informed consent to participate in this study.

## Author Contributions

FR contributed to the study design and data analysis. AF contributed to the study design and management of data gathering. SG, LD, ALu, and MB contributed to the data gathering. MC supervised data analysis and reviewed the manuscript. ALe contributed to the study design, coordinated the research team, and reviewed the manuscript.

## Funding

The study was financed by the Montreal Regional Health and Social Services Agency (ASSSM), as work package of a re-organization process of the mental health and social services system.

## Conflict of Interest

The authors declare that the research was conducted in the absence of any commercial or financial relationships that could be construed as a potential conflict of interest.
